# Sex-Specific Signaling in the Blood–Brain Barrier Is Required for Male Courtship in *Drosophila*

**DOI:** 10.1371/journal.pgen.1003217

**Published:** 2013-01-24

**Authors:** Valbona Hoxha, Chamala Lama, Peter L. Chang, Sumit Saurabh, Naiya Patel, Nicole Olate, Brigitte Dauwalder

**Affiliations:** 1 Department of Biology and Biochemistry, University of Houston, Houston, Texas, United States of America; 2 Department of Biological Sciences, University of Southern California, Los Angeles, California, United States of America; New York University, United States of America

## Abstract

Soluble circulating proteins play an important role in the regulation of mating behavior in *Drosophila melanogaster*. However, how these factors signal through the blood–brain barrier (bbb) to interact with the sex-specific brain circuits that control courtship is unknown. Here we show that male identity of the blood–brain barrier is necessary and that male-specific factors in the bbb are physiologically required for normal male courtship behavior. Feminization of the bbb of adult males significantly reduces male courtship. We show that the bbb–specific G-protein coupled receptor *moody* and bbb–specific Go signaling in adult males are necessary for normal courtship. These data identify sex-specific factors and signaling processes in the bbb as important regulators of male mating behavior.

## Author Summary

Complex behaviors such as mating behavior are controlled by the brain. Ensembles of brain cells work in networks to ensure proper behavior at the right time. While the state of these cells plays an important role in whether and how the behavior is displayed, information from outside the brain is also required. Often, this information is provided by hormones that are present in the circulating fluid (such as the blood). However, the brain is protected by a layer of very tight cells, the so-called blood–brain barrier, that keeps unwanted molecules out. So how then do hormones and other regulatory factors “talk” to the brain? We are studying this question by examining the mating behavior of males of a model organism, the fruit fly *Drosophila melanogaster*. We have found that the blood–brain barrier cells themselves contain male-specific molecules that play an important role. When they are absent, courtship behavior is compromised. We have also identified how outside factors talk to the brain: by using a cellular signaling protein and a particular signaling pathway. Together they are well suited to pass on outside information to the brain network that regulates mating behavior.

## Introduction

Male courtship in *Drosophila melanogaster* consists of a series of consecutive behavioral steps that are well characterized (for reviews see [1]–[3]) and include orientation towards the female, tapping of the female's abdomen with the male's forelegs, performing of a courtship “song” by wing extension and vibration, licking of the female's genitalia, attempted copulation and copulation. The behavior is controlled by the master regulatory genes of the general somatic sex determination pathway *Sxl*, *tra*, *dsx* and *fru* [4]–[6]. A cascade of sex-specific alternative splicing results in males that express the transcription factors DSXM and FRUM, and females expressing DSXF (the FRU protein is not made in females). Both FRU and DSX are expressed in defined subsets of neurons in the brain and abdominal ganglia, with expression overlapping in some of these neurons. A number of experiments have demonstrated that both *dsx* and *fru* are required for normal male courtship behavior ([7]–[12]; reviewed in [13]–[16]) and several recent studies have identified *dsx* and *fru* neuronal projections that form putative circuits that confer the competence for male courtship behavior [10], [17]–[19]. While *fru* and *dsx* neurons are indispensible for male courtship, another, non-neuronal tissue has emerged as an important regulator: the fat body. The fat body (which is often compared to the mammalian liver) is a secretory tissue that surrounds the brain, the abdominal ganglia and the abdominal organs. Among other important aspects it functions in metabolism, energy storage, immune function and yolk protein synthesis in females. It also produces sex-specific factors that are required for full male courtship behavior [20], [21]. When, by genetic means, the fat body is made female in an otherwise normal adult male, courtship behavior is strongly reduced, indicating that it produces sex- specific factors [22]. These experiments are feasible because sex determination in flies is mostly cell-autonomous. In agreement with this finding, several other screens for sex-specifically expressed genes in fly heads, and for genes that are controlled by *dsx* and *fru*, resulted in the identification of genes that are expressed in the fat body [23]–[26]. The fat body secretes proteins into the hemolymph, the open circulatory system of the fly. Takeout, one of the male-enriched fat body proteins with a role in male courtship, has been shown to be secreted into the hemolymph and act as a secreted protein [22]. This is likely also the case for other sex specific fat body proteins. How such hemolymph proteins interact with the brain circuits that control courtship is unknown. Since flies possess a blood–brain barrier (bbb), hemolymph proteins do not have unrestricted access to brain neurons. Nothing is known about the mechanisms by which these proteins negotiate the blood–brain barrier. The *Drosophila* bbb consists of two glial cell layers that ensheathe the entire brain, ventral ganglion and peripheral nerves. The inner or subperineurial glia (SPG) cell layer is situated next to the neuronal cell bodies and forms the tight barrier that is characteristic of vertebrate and invertebrate blood–brain barriers [27]–[30]. The vertebrate bbb is formed by tight junctions between blood endothelial cells [31]. In contrast, in *Drosophila*, septate junctions form the basis of the barrier, similar to mammalian myelinating glia at the paranodal junctions. Several studies have identified important proteins that are required for the development of a tight and functional barrier, several of which are evolutionarily conserved [29], [30], [32]–[35]. While insight into the development of the *Drosophila* bbb has been gained, very little is still known about the adult bbb and the mechanisms that underlie its function (for a review see [36]). Interaction of hemolymph components with the brain will likely involve specific receptors, transporters and signaling events. A *Drosophila* ABC transporter Mdr65 that functions similarly to the mammalian human MDR1/Pgp has been described [37]. Furthermore, the putative GPCR *moody* that is specifically expressed in the SPG barrier cells has been shown to mediate the behavioral response to ethanol and cocaine independent of its developmental function in barrier set up [28].

In order to approach the question how sex-specific circulating factors communicate/pass through the bbb in order to exert their function, we examined whether the sexual identity of the bbb itself might be required, and whether *moody* signaling in the bbb is a component in the regulation of courtship behavior. We report here that the bbb contains sex specific factors that are important for male courtship, and that *moody* and Go protein signaling in the bbb are important regulators of this behavior.

## Results

### Feminization of the blood–brain barrier reduces male courtship behavior

**Figure 1:**
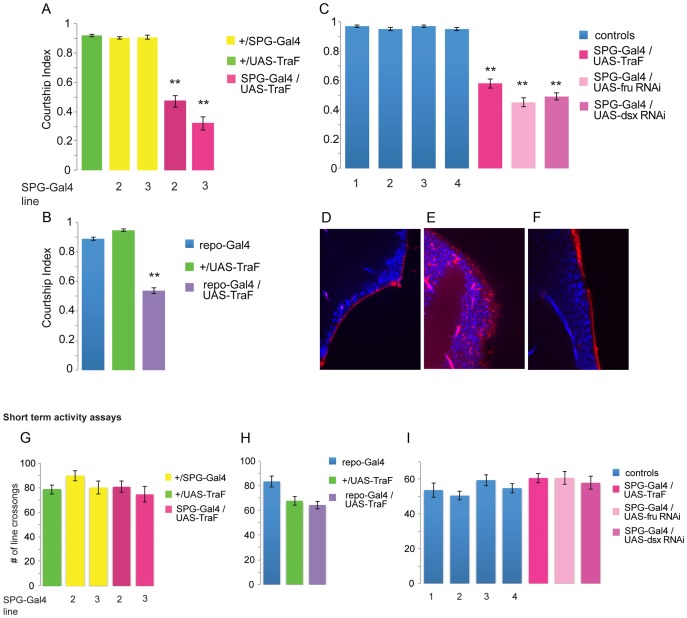
The sexual identity of bbb cells is important for courtship. Graphs show the courtship index CI (fraction of time males spend courting during the observation period) ± Standard Error of the Mean (SEM) of the indicated genotypes (a–c), or the performance of males in a control activity assay (# of line crossings ± SEM) (g–i). N = 20 for all genotypes. Data were analyzed by ANOVA followed by Bonferroni multiple comparisons (p<0.0001). Indices that are significantly different from the controls are marked by asterisks. a) Expression of *UAS-TraF* using two independent bbb-specific *SPG-Gal4* drivers on the third (3) or second (2) chromosome significantly reduces male courtship. b) Expression of *UAS-TraF* using the glial driver *repo-Gal4* significantly reduces male courtship. c) Expression of *UAS-fru RNAi*, *UAS-dsx RNAi* and *UAS-TraF* in the bbb by *SPG-Gal4*(3) similarly reduce male courtship in comparison to controls. The controls are 1) *+/UAS-dsx RNAi*, 2) *+/UAS-fru RNAi* 3) *+/SPG-Gal4*, 4) *+/UAS-TraF*. d-f) The blood–brain barrier integrity is not compromised in *SPG-Gal4/UAS-TraF* mutants. Flies were injected with 10 kDa TR-dextran (red) and dye penetration into or exclusion from the brain was observed by confocal microscopy. The brain nuclei are stained with DAPI. A tight bbb is observed by a demarcated red line on the surface of the brain indicating exclusion of TR-dextran from the brain in the *CS* control (d) and *SPG-Gal4/UAS-TraF* (f) males. e) For comparison, dye penetration is shown in *moody* null flies which have a compromised bbb [28]. g, h, i) Activity assays for the genotypes assayed in a, b, c). While there is some variation among controls, likely due to genetic background, none of the mutant courtship phenotypes can be attributed to a difference in activity in comparison to the controls.

To examine whether the bbb has a sex-specific role in male courtship behavior, we used the sub-perineurial glia specific Gal4 driver *SPG-Gal4* [30] to express a *UAS- TraF* transgene. TraF is a female-specific regulator of the sex determination cascade and expression of TraF has been shown to specifically feminize the cells in which it is expressed [38]–[40]. We used two different SPG-Gal4 lines with insertions on the second or third chromosome, respectively, to express TraF (gifts from R. Bainton). Males whose bbb layer was feminized court females significantly less than the wild-type control males (Figure 1a). Their overall courtship index (CI) was significantly reduced. The CI is the fraction of time a male spends performing any of the steps of the courtship ritual within the observation period. When we quantified individual courtship steps, we found that latency (the time to first orientation towards the female) was the same in control and experimental animals. This indicates that the mutants are not deficient in their perception of the female and her pheromones. In contrast, the fraction of time spent extending a wing to perform the courtship song and the number of copulations were significantly reduced, in agreement with the overall reduction in courtship (Table 1). These data show that males with feminized blood–brain barrier are capable of all steps of courtship, but perform them with reduced probability. This is not due to locomotion defects, since males with feminized bbb perform indistinguishably from control flies in a short term activity assay [41] (Figure 1g). In this assay, individual males are placed in a courtship chamber and the number of time they cross a drawn line is counted. To corroborate that the observed courtship reduction is caused by the feminization of glial cells, we used *repo-Gal4*, a driver that is expressed not just in the bbb but generally in glial cells [42]. We observed a similar reduction, confirming that the male identity of glial cells is important for male courtship (Figure 1b, 1h). This would predict that a similar effect should be observed if these cells were made “less male”. Since TraF acts through its downstream targets *fru* and *dsx* we next examined the effect of expressing RNAis that target these transcripts in bbb cells. Indeed, *SPG-Gal4/UAS-fruRNAi* and *SPG-Gal4/UAS-dsxRNA*i males showed similar reductions as *SPG-Gal4/UAS-TraF* animals (Figure 1c, 1i). This suggests that FRU and DSXM both have a role in regulating sex-specific molecules in the bbb. It also argues that the effect of TraF is not due to merely overexpressing female-specific DSXF.

**Table 1:** Individual courtship elements in *SPG-Gal4/UAS-TraF* males.

	*+/SPG-Gal4*	*+/UAS-TraF*	*SPG-Gal4/UAS-TraF*
**Courtship Index**	0.90±0.02	0.91±0.018	0.31±0.056**
(All Steps of Courtship)			
**Latency** (s)	14.9±4.94	14.8±5.22	16.1±3.7
(Time to first orientation toward female)			
**Wing Extension Index**	0.72±0.04	0.77±0.04	0.12±0.04**
(% of total observation time)			
**Wing Extension**	0.79±0.027	0.85±0.041	0.33±0.076**
(% of total courtship time)			
**Attempted Copulations**	2.8±2.25	4.3±1.63	0.6±0.33

Individual courtship steps in a standard courtship assay were analyzed for males of the indicated genotypes paired with a wild-type virgin female. Values are mean ± SEM. Latency: The time to first orientation toward the female is indicated. For wing extension, the relative time engaged in this behavior relative to the total time spent courting was calculated. For attempted copulation, the total number of events is given. Copulation was not scored since the females were only a few hours old and resisted copulation.

** Values that were significantly different from those of the control flies.

An important question that arises from these results is whether the feminization of the bbb affects the blood–brain barrier permeability. To address this question we performed a dye penetration assay [28], [37]. An intact bbb will exclude a 10 kDa Texas-red-coupled dextran (TR-dextran) molecule from the brain, whereas in mutants with a defective bbb, the dye will penetrate through the bbb (Figure 1e). We injected 10 kDa TR-dextran into adult males that prior to the injections were raised and treated identical to the males in the courtship assay. After a 24 h recovery period, fly brains were dissected and dye penetration into the brain was examined by confocal microscopy (Figure 1d–1f). TR-dextran was efficiently excluded from the brain of males with feminized bbb (Figure 1d), indistinguishable from the CS wild-type control (Figure 1f). These results suggest that feminization of the sub-perineurial layer of the bbb does not affect blood–brain barrier permeability in an obvious way, but rather affects sex-specific physiological processes that are required for male courtship behavior.

**Figure 2:**
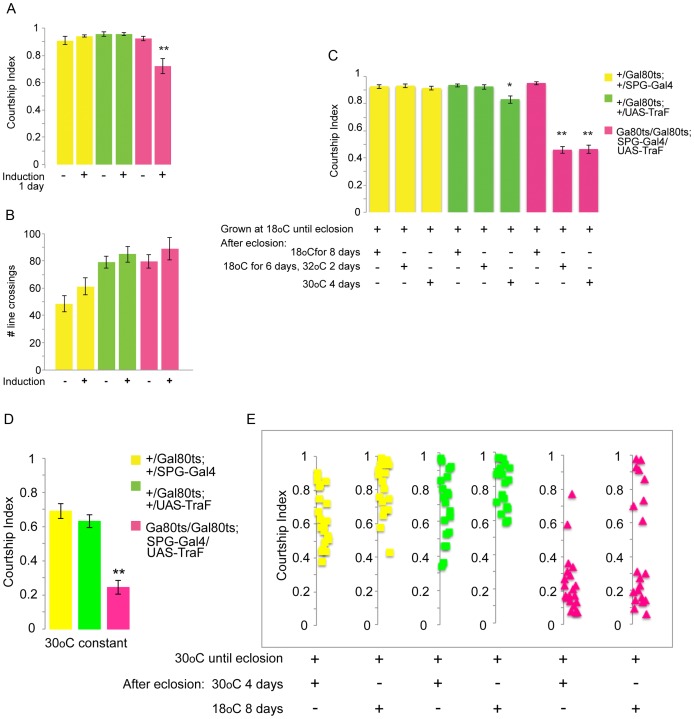
Conditional adult expression of *UAS-TraF* by *SPG-Gal4(3)* reduces male courtship. Graphs show the courtship index CI (fraction of time males spend courting during the observation period) ± SEM of the indicated genotypes, or the performance of males in a control activity assay (# of line crossings ± SEM). N = 20 for all genotypes unless indicated otherwise. Data were analyzed by ANOVA followed by Bonferroni multiple comparisons (p<0.0001). Indices that are significantly different from the controls are marked by asterisks. *UAS-TraF* expression is restricted by the presence of *tubP-Gal80^ts20^* and *tubP-Gal80^ts10^* at 18°C (induction −). a) Placement of 5-day-old males at 32°C for 16 hours (induction +) releases the Gal80 inhibition and leads to the expression of TraF (N = 10). b) The mutants have activity levels similar to the controls (N = 15). c) Induction for longer time periods at either 30°C or 32°C lowers the courtship index further. Flies were grown at 18°C until eclosion and either kept at 18°C for 8 days until testing (induction −), or kept at 18°C for 6 days and then shifted to either 32°C for 2 days, or to 30°C for four days, as indicated in the table below the graph (N = 20). d) Courtship index of flies shifted from 25°C to 30°C as wandering third instars for the rest of development and throughout adult life prior to testing. e) All flies were shifted from 25°C to 30°C as wandering third instars for the rest of development. Upon eclosion, they were either down-shifted to 18°C for 8 days and then tested, or kept at 30°C until testing on day 4, as indicated in the table below the graph.

Because the sub-perineurial layer is formed during blood–brain barrier development [29], but also has a physiological role in adults [28], the observed reduction in male courtship behavior could be a result of developmental effects, an effect on the adult physiological function of the bbb, or both. To distinguish between these possibilities, we added a temperature sensitive *tubP-Gal80^ts^* transgene [43] to induce feminization of the sub-perineurial layer exclusively in the adult. Males were raised at 18°C and after eclosion kept at 18°C. At that temperature, the Gal80^ts^ inhibitor is active and inhibits Gal4 from activating UAS-TraF. Mature adult males were then transferred to 32°C or 30°C before testing in the courtship assay. At 30°C and 32°C, Gal80^ts^ becomes inactive, Gal4 is active and TraF is induced [43]. Control animals were continuously kept at 18°C (uninduced controls). The results are shown in Figure 2. Control genotypes at the uninduced (18°C) and induced temperatures courted normally. Uninduced *Gal80^ts20^/Gal80^ts10^*; *SPG-Gal4/UAS-TraF* males courted females normally. In contrast, induced *Gal80^ts20^/Gal80^ts10^*; *SPG-Gal4/UAS-TraF* males showed a reduction in courtship behavior. This effect was dependent on the length of induction. Induction at 32°C for 16 hours (Figure 2a) reduced courtship to a lesser extent than induction for 48 hours (Figure 2c). Extended induction at 30°C for four days did not reduce courtship further (Figure 2c) (30°C was chosen to reduce the impact of the extended heat-shock period). As observed in the feminization experiments not using Gal80, all experimental males were capable of performing all courtship steps, but they performed them less frequently. The reduction in the extended time-induced males was close to the reduction observed in the absence of Gal80, indicating that most of the observed effect was due to the effect of feminization during the mature adult stage. To explore this further, we examined *Gal80^ts20^/Gal80^ts10^*; *SPG-Gal4/UAS-TraF* males that spent most of their development at 30°C. Due to lethality of the higher temperature during earlier stages, we moved the flies from 25°C to 30°C on day 5, when third instar wandering larvae began climbing up the wall of the vial. The critical time period for behavioral sex-determination has been shown to lie in pupal stages [4]. As shown in Figure 2d, extended heat conditions lowered overall courtship somewhat in the controls. In the *Gal80^ts20^/Gal80^ts10^*; *SPG-Gal4/UAS-TraF* males the courtship index was reduced similarly to the reduction in the *SPG-Gal4/UAS-traF* males shown in Figure 1a, relative to control flies. However, these males rarely showed courtship behaviors beyond their initial orientation to the female. These results may indicate a developmental component when feminization occurs throughout extended stages. We next tested whether males that had spent most of their development at 30°C could be rescued by shifting them back to 18°C after eclosion. As shown in Figure 2e, a fraction of these flies was rescued and showed wild-type courtship scores (8 out of 20). The rest, rather than being partially rescued, did not improve. This “all or none” rescue may indicate a threshold effect. However, the males that did show rescue demonstrate the importance of correct male identity of the bbb cells in the adult. Taken together, our experiments demonstrate a significant sex-specific role of the bbb in the control of male courtship and suggest that sex-specific molecules in the bbb play a crucial physiological role in the regulation of courtship.

### The blood–brain barrier–specific Moody G-protein-coupled receptor (GPCR) is required for male courtship behavior

**Figure 3:**
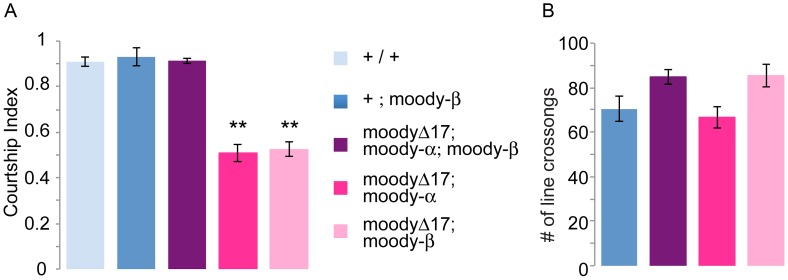
*moody-α* and *moody-β* mutants have courtship defects. The graph shows the courtship index CI (fraction of time males spend courting during the observation period) ± SEM of the indicated genotypes, or the performance of males in a control activity assay (# of line crossings ± SEM). N = 20. Data were analyzed by ANOVA followed by Bonferroni multiple comparisons (p<0.0001). Indices that are significantly different from the controls are marked by asterisks. Δ17 is a *moody* null mutant with a leaky bbb. The bbb barrier defect can be rescued by the addition of either the Moody-α or Moody-β protein isoform [28]. a) In contrast, despite the intact bbb, expression of only one isoform is not sufficient for normal courtship. *moody-α* and *moody-β* mutants have a courtship defect. When both the Moody-α and Moody-β isoforms are present in *moody* Δ17 mutants, courtship is restored to wild-type levels. b) The activity of the mutants is not different from that of control flies.

Signaling molecules are likely candidates for mediating the courtship role of the bbb, but very little is known about proteins that are expressed in this layer. Among them, *moody* is a good candidate for such a role. *moody* encodes a putative GPCR that has been shown to be specifically expressed in the sub-perineurial glia (SPG) layer of the blood–brain barrier. While *moody* is required developmentally for the formation of a tight bbb [29], it has an independent role in the response of adult animals towards ethanol and cocaine [28]. The gene encodes two different protein isoforms, Moody-α and Moody-β that are formed by differential alternative splicing [28]. *moody* null mutant animals (*moodyΔ*17) are lethal with a leaky bbb, although a few escapers can survive into adulthood. Addition of either a *moody-α* or a *moody-β* encoding transgene is sufficient to rescue the lethality and the leakiness of the bbb [28]. However, despite their intact bbb, *moodyΔ*17; *moody-α* or *moodyΔ*17; *moody-β* flies are mutant in their sensitivity to ethanol and cocaine, demonstrating that both protein isoforms are required for the behavior [28]. We tested the courtship of *moodyΔ*17; *moody-α* and of *moodyΔ*17; *moody-β* flies and found that their courtship was significantly reduced (Figure 3a). However, the reduced courtship behavior was rescued when both transgenes were present. The mutant genotypes did not affect short term activity, as shown in Figure 3b. We conclude from these experiments that *moody-α* and *moody-β* are both required in the bbb for normal courtship behavior.

**Figure 4:**
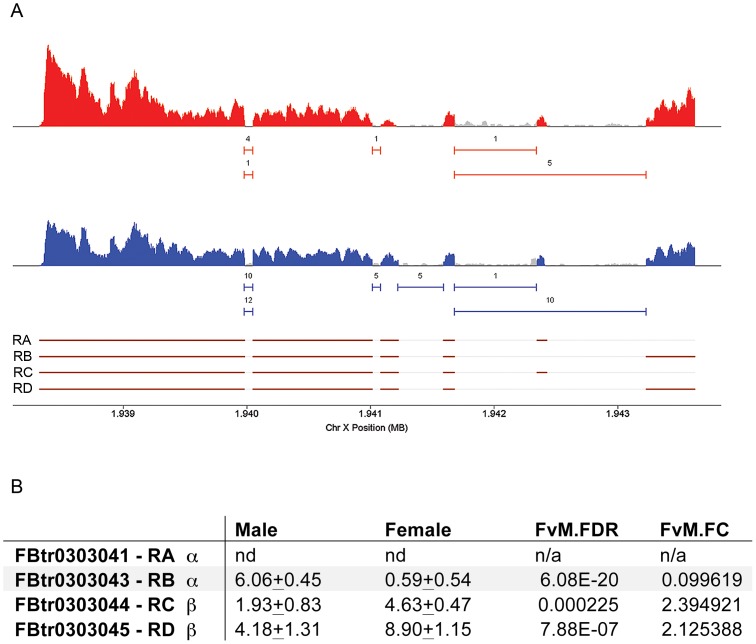
*moody* transcript isoforms are expressed differently in males and females. a) Coverage plots, junction plots and gene models [44] for *moody* are shown. Coverage plots for exon sequences are shown with peaks in red (females) and blue (males) indicating coverage from RNA from females and males, respectively; grey indicates non-exonic gene regions as annotated by Flybase. Junction plots are shown as solid horizontal lines beneath the coverage plots. The number above each line indicates the number of sequence reads that span a junction. All numbers are based on 1 million mapped reads. Flybase gene models are shown at the bottom of the panel with exon regions shown in brown. b) Of the four previously identified isoforms (http://flybase.org/reports/FBgn0025631.html), one (FBtr0303041-moody RA) was not detected in adult heads. FBtr0303043 (moody-RB) which encodes an alpha isoform is significantly enriched in males in comparison to females, whereas FBtr0303044 (moody-RC) and FBtr0303045 (moody RD) which encode beta isoforms are enriched in females in comparison to males. F: Female; M: Male; FDR: False discovery rate; FC: Fold change.

When we analyzed RNAseq data generated from adult male and female heads [44] we found that the *moody* isoforms are expressed differently in the two sexes (Figure 4). There are four *moody* RNA isoforms that differ in their use of two transcription start sites and by differential splicing (by 2 nucleotides) of one of the introns that generates either the α or β protein isoform [28]. Of the four previously identified isoforms (http://flybase.org/reports/FBgn0025631.html), one (FBtr0303041-moody-RA) was not detected in adult heads. FBtr0303043 (moody-RB) which encodes an alpha isoform is significantly enriched in males, whereas FBtr0303044 (moody-RC) and FBtr0303045 (moody-RD) which encode beta isoforms are enriched in females. However, our courtship data presented in Figure 3a show that both α and β isoforms are required for courtship in males.

### G_o_ signaling in the blood–brain barrier is required for male courtship behavior

Since Moody is a putative GPCR that potentially signals through G proteins, we next examined the importance of G-protein signaling in the bbb for male courtship behavior. We tested mutant flies for each of the known G- proteins in *Drosophila*: Go, Gs, Gi, and Gq. In addition, we tested a RNAi mutant of a fifth Gα subunit (*concertina*) that has been shown to be present in the fly genome [45]. It has been reported that Go signaling is required for blood–brain barrier insulation during development [29] and G-protein mediated signaling is required throughout development. Therefore, to circumvent developmental lethality, we conditionally expressed mutant forms of the proteins only in adult mature males using the Gal80^ts^ system.

**Figure 5:**
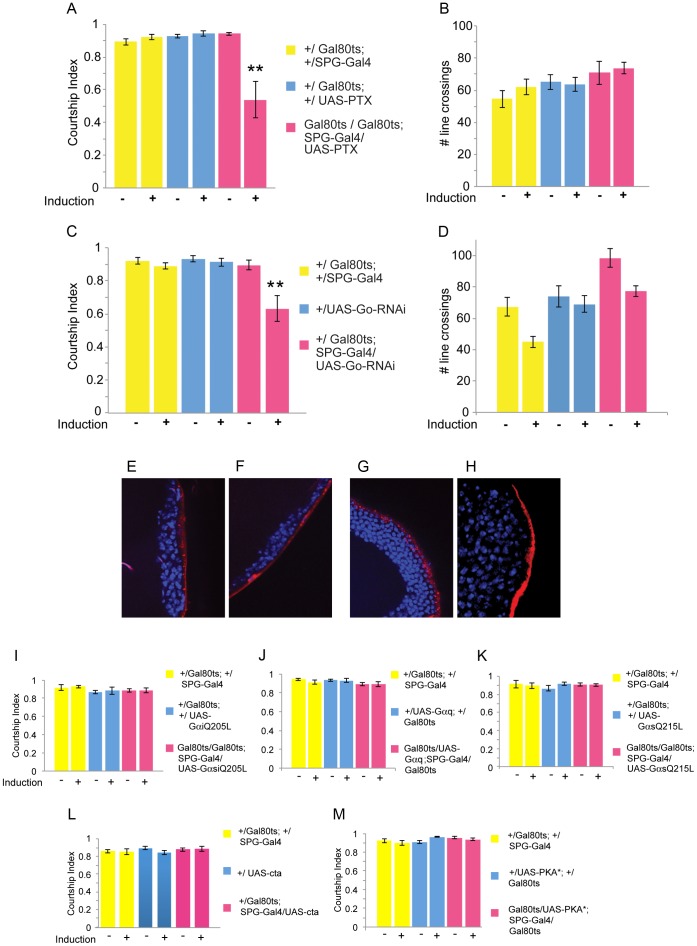
Go signaling is required for courtship. Graphs show the courtship index CI (fraction of time males spend courting during the observation period) ± SEM of the indicated genotypes, or the performance of males in a control activity assay (# of line crossings ± SEM). N = 10. Data were analyzed by ANOVA followed by Bonferroni multiple comparisons (p<0.0001). Indices that are significantly different from the controls are marked by asterisks. a) Conditional adult expression of *UAS-PTX* by *SPG-Gal4* significantly reduces male courtship. The S1 subunit of PTX from *B. pertussis* specifically inhibits Go in *Drosophila*. *UAS-PTX* expression is restricted by the presence of *tubP-Gal80^ts^* at 18°C (induction −). Placement of 5-day-old males at 32°C for 16 hours (induction +) releases the inhibition and leads to the expression of PTX. b) The mutants have activity levels similar to the controls. c) Conditional adult expression of *UAS-Gαo-RNAi* by *SPG-Gal4* significantly reduces male courtship (this genotype was induced for 3 days); the mutants have activity levels similar to the controls (d). e–h) The blood–brain barrier does not show leakage in *SPG-Gal4/UAS-PTX* (PTX) (e, f) *and SPG-Gal4/UAS-Gαo-RNAi* (RNAi) (g, h) mutants. Uninduced (18°C; e, g) and induced (32°C; f, h) mutant males that were treated identically to the ones tested in courtship were injected with 10 kDa TR-dextran (red) and dye distribution was observed by confocal microscopy. Brain nuclei are stained with DAPI. A tight bbb is observed as a demarcated red line on the surface of the brain indicating exclusion of TR-dextran from the brain. i–l) Conditional expression of dominant mutant versions or RNAi targeting Gαi, Gαq, Gαs and *concertina* (*cta*) in the bbb using SPG-Gal4(3). 5-day-old males were induced by placement at 32°C for 16 hours. m) Similarly, conditional expression of PKA* (a persistently activated PKA catalytic subunit) in the bbb did not affect courtship.

To specifically inhibit Go we used the bbb specific SPG-Gal4 to drive expression of pertussis toxin (PTX) (Figure 5a, 5b). The S1 subunit of PTX from *B. pertussis* specifically ADP-ribosylates vertebrate G(i/o/t) proteins, resulting in their inability to bind to activated GPCRs [46]. Flies do not have Gt, and their Gi lacks the site for ADP ribosylation. Thus, PTX in flies is specifically inhibiting Go [47]–[49]. After the induction of PTX expression in the SPG layer for 12 hours at 32°C, we observed a significant reduction in male courtship behavior (Figure 5a). A breakdown of courtship into the different steps indicates that in the mutants latency is normal, but the probability that a male will re-initiate and sustain courtship beyond the first steps is lowered, although all steps, including copulation, can be carried out (Table 2). In addition, flies with induced PTX expression are normal in the short-term activity assay (Figure 5b). As a complementary approach to the inhibition of Go signaling by PTX, we conditionally reduced Go signaling in bbb cells by Gαo-RNAi. In agreement with the PTX results, Gαo reduction in adult mature males reduced male courtship (Figure 5c). Short-term activity of the mutants was normal (Figure 5d). To examine whether interference with Go signaling in adult males affects the integrity of the bbb, we performed TR-dextran dye penetration assays. Conditional adult expression of PTX or Go-RNAi in the blood–brain barrier does not affect the insulating properties of the blood–brain barrier (Figure 5e–5h). Conditional bbb expression of dominant mutant forms of Gs and Gi [50], [51], and of *Gq-RNAi* and *cta-RNAi* had no effect on courtship (Figure 5i–5l), although the mutant Gs and Gi proteins have previously been shown to be active and affect development [29], [50], [52]. We also did not observe a courtship phenotype when we disrupted PKA signaling, a potential downstream effector, by expressing PKA*, a dominant persistently active mutant catalytic subunit [53]. Taken together, our results strongly suggest that Go signaling in the bbb is physiologically required for the regulation of normal male courtship behavior.

**Table 2:** Individual courtship elements in *SPG-Gal4/UAS-PTX* males.

	*+/Gal80ts10*; *+/SPG-Gal4*		*+/Gal80ts20*; *+/UAS-PTX*		*Gal80ts10/Gal80ts20*; *SPG-Gal4/UAS-PTX*	
	−	+	−	+	−	+
**Courtship Index**	0.91±0.02	0.92±0.02	0.92±0.02	0.95±0.02	0.94±0.01	0.61±0.11**
(All Steps of Courtship)						
**Latency** (s)	4.1±2.08	6.1±2.73	4.7±1.45	7.9±2.8	7±2.77	8.86±2.53
(Time to first orientation toward female)						
**Wing Extension Index**	0.67±0.03	0.65±0.03	0.60±0.04	0.57±0.046	0.59±0.024	0.32±0.085**
(% of total observation time)						
**Wing Extension**	0.74±0.03	0.70±0.02	0.65±0.04	0.59±0.05	0.54±0.02	0.48±0.08
(% of total courtship time)						
**Attempted Copulations**	0.8±0.49	1.6±0.62	1.9±0.96	0.7±0.26	1.5±0.66	1±0.41

Individual courtship steps in a standard courtship assay were analyzed for males of the indicated genotypes paired with a wild-type virgin female. Values are mean ± SEM. Latency: The time to first orientation toward the female is indicated. For wing extension, the relative time engaged in this behavior relative to the total time spent courting was calculated. For attempted copulation, the total number of events is given. Copulation was not scored since the females were only a few hours old and resisted copulation.

** Values that were significantly different from those of the control flies.

## Discussion

The blood–brain barrier is an important selective interface between circulating factors and the brain. We show here that the bbb also plays a crucial role in the regulation of male courtship behavior in *Drosophila*. When the bbb is feminized in an otherwise normal male animal, the courtship index drops significantly, indicating the presence of male-specific factors and processes in these cells. Importantly, while some of these sex-specific factors may affect sex-specific development of the bbb, our results demonstrate that feminization of the bbb exclusively in the adult is sufficient to reduce male courtship. Thus, male-specific factors are physiologically required in courting males. It is worth noting that the integrity of the bbb was not affected by feminization or by any other of our manipulations using a standard approach to examine bbb barrier function, although we can not rule out small defects. Therefore, the observed effects support the interpretation that feminization affects physiological sex- specific processes within the bbb. We show here that *moody* GPCR signaling is one of these processes. Normal courtship requires both *moody* isoforms, α and β, similar to the previously reported response to alcohol and cocaine. As has been described, *moody* appears to have two distinct roles: While either one of the *moody* isoforms is sufficient for a functional and intact barrier, both isoforms are required for adult signaling processes. Our RNA sequencing data suggest that the two isoforms are not present in equal abundance and that the ratio of the two isoforms is sex-specifically regulated. It is not clear at present why two forms of the *moody* protein are required in behavior. It is unlikely that a strict stoichiometric ratio of the two isoforms is required, since we have observed normal courtship in wild-type flies that express additional Moody-β (+/moody−β in Figure 3a). The two isoforms differ in their intracellular domain, which could indicate that they interact with different effector molecules that are both contributing to the behavioral response. We have observed courtship defects when either isoform is missing, indicating that both forms have a role in regulating courtship. Interestingly, we have observed that the ratio of the two isoforms is under the control of the sex-specific splicing factor TraF [44], raising the possibility that the *moody* pre-mRNA is a target for splicing regulation by TraF or one of its downstream effectors. It will be of interest to identify other sex-specific factors in the bbb and examine their contribution to the regulation of male courtship.

We did not observe a courtship phenotype when we expressed dominant mutants for Gs and Gq that have been shown to act as dominant negative mutations in developmental processes [50]. Likewise, expression of Gi-RNAi or *concertina-RNAi* did not result in reduced courtship either. This suggests that these G proteins do not play a significant role in courtship in this layer. In contrast, we have observed courtship defects when Go signaling was compromised. We have employed two approaches to show that the heterotrimeric protein Go is required for male courtship behavior: inhibition by PTX and mRNA reduction by RNAi. The S1 subunit of PTX from *Bordetella pertussis* catalyzes the transfer of an ADP-ribose onto the Gα subunit of the heterotrimeric G protein. In contrast to mammals, where PTX inhibits both Go and Gi, in *Drosophila* PTX is a specific inhibitor for Go, since the only Gi present (G(i)65A) does not contain the PTX recognition site. PTX will only ribosylate heterotrimers (not individual alpha subunits), and the consequence of this ribosylation is inhibition of the heterotrimer activation [46], [54]. The inhibition of Go signaling by PTX is therefore very specific; since the ADP-ribosylated Go heterotrimers cannot be activated, they do not generate ectopic Gβγ subunits, nor do they sequester free Gβγ subunits away from other Gα subunits. Conditional induction of PTX only in adult mature flies, as well as conditional adult reduction of Gαo by RNAi reduced male courtship. This demonstrates that physiological signaling through Go is an important signaling pathway that regulates courtship in the bbb. Given these findings it is likely that *moody* signals through Go to exert its function in courtship. In embryos, Go, Gi, *moody* and *loco* mutations each disrupt the formation of the bbb [29] and lead to bbb leakiness, as shown by dye penetration. In contrast, we did not observe dye penetration in the PTX and Go-RNAi mutants that we generated, further evidence that the developmental and physiological roles of *moody* and Go signaling differ in their mechanisms.

We do not know what the downstream pathways are that are mediating the Go action. Few Go effectors have been demonstrated and its α or βγ subunits could be mediating the signal. In many cases in vertebrates it is the βγ subunits that are responsible for actuating signaling. In neurons, presynaptic voltage-gated Ca^2+^ channels have been shown to represent an effector for Go [55]. Studies of the role of Go in learning and memory in *Drosophila* have suggested that Go signaling does not occur through the *rut* adenylyl cyclase [47]. Signaling through Go is not generally thought to occur through PKA, consistent with our finding that disruption of PKA signaling in the bbb did not affect courtship. It is unknown whether potential downstream signaling molecules like *loco*, Gγ13F or PKC are sex-specifically expressed in the blood–brain barrier and might have a courtship role in this layer. In whole heads, PKC98E is male-preferentially expressed [44]. It is unknown what the ligand is for Moody and it remains to be seen what the exact role is for *moody* bbb signaling in courtship. Hemolymph factors that influence courtship could conceivably do so by initiating signaling pathways at the bbb, or by passage and transport through the bbb. Moody could be playing a role in signaling, as well as through a possible effect on transport, perhaps in processes similar to its previously demonstrated effects on the actin cytoskeleton during development [29].

Here we have demonstrated that sex-specific molecules in the bbb are important regulators of male courtship behavior in *Drosophila*. The Moody GPCR and Go signaling in this layer are an important part of this regulation. It will be of importance to identify the ligand(s) and downstream signaling events that ultimately interact with the brain circuits that control male courtship behavior.

## Materials and Methods

### Fly strains

All flies strains were reared on standard corn meal/sugar-based medium at room temperature under non-controlled light conditions, except for *Gal80^ts^* flies that were grown at 18°C and induced as adults at 32°C or 30°C as indicated. *TraF* is *w^1118^*; *P{UAS-tra.F}20J7* [*39*]; *SPG-Gal4/TM3*, *SPG-Gal4/CyO*, *Δ17/FM6K*; *Δ17/FM6K;α- moody/+* and *Δ17/FM6K*; *β- moody/+* and *Δ17/FM6K;α- moody/+*; *β- moody/+* were a gift from Dr. Roland Bainton (University of California at San Francisco, San Francisco, California). *y, repo-Gal4* on X was a gift from Takeshi Awasaki (University of Massachusetts, Worcester, Massachusetts) [27]; Prior to using the flies in courtship crosses, we removed the *y* mutation by recombination. *w; UAS-G-salpha60A.Q215L}16/TM3* (BL6490) [51] and *UAS-concertina RNAi TRiP.JF01607}attP2* (BL 31132) were obtained from the Bloomington *Drosophila* stock center; The strains *w; UAS-G- ialpha65A.Q205L}16* [50]; *P{UAS-PTX}16,ry506/TM3*; *w*; *Sco/CyO*; *tubP-Gal80^ts2^*, *w*; *tubP-Gal80^ts10^*, *w; tubP-Gal80^ts20^* were a gift from Dr. Gregg Roman (University of Houston, Houston, Texas). The Go RNAi strain *UAS- CG2204 RNAi^19124^* (Transformant ID :19124) was obtained from the VDRC stock collection, Vienna.

### Gal80^ts^ experiments

For *Gal80^ts^* experiments, control and experimental flies were raised at 18°C. Virgin males were collected at eclosion and kept for 5–8 days at 18°C. Matured flies were then placed at 32°C or at 30°C for the times indicated in the text. For some of the temperature shift experiments, crosses were placed at 25°C for 5 days, when third instar wandering larvae began climbing up the walls. At that time the vials were moved to 30°C. Freshly eclosed males were individually put in vials and matured at the temperatures and for the time spans indicated in the figures. Only flies that eclosed within the first 24 hours were used. All flies were let rest for 1–2 hours at RT prior to the behavioral assay. Non-induced controls from 18°C were subjected to the same resting period of 1–2 hours at room temperature before testing.

### Behavioral assays

The courtship assay and activity assay were performed as previously described [20]. In short, males were placed in a plexiglas “mating wheel” (diameter 0.8 cm), together with a 2–4 hrs old Canton-S virgin female. The courtship index was calculated as the fraction of time the male spent displaying any element of courtship behavior (orienting, following, wing extension, licking, attempted copulation, copulation) within a 10 minute observation period [6]. Short-term activity assays were performed as previously described [41]. Individual males were placed into the “mating wheel” containing a filter paper with a single line dividing the chamber in half. After 2–3 minutes of acclimation time, the number of times the male crossed the center line within the three minute observation time was counted.

Each graph represents a full set of control and experimental genotypes that were grown, collected and aged in parallel. In each behavioral session, equal numbers of all genotypes were tested. The number of tested flies was equal for all genotypes in an experiment.

### Dye injection assay

We injected 10 kDa TR-dextran into adult males that prior to the injections were raised and treated identical to the males in the courtship assay. The animals were anesthetized on ice and injected with 2.5 mM 10 kDa Texas-Red conjugated dextran (Invitrogen D-1863) in H_2_O as previously described [28] with slight modifications. Immobilized flies were kept cold by ice packs during dye injection. 0.1–0.3 µl of dye was microinjected into the scutellum of the fly. After injection flies were allowed to recover overnight. Flies were anesthetized on ice and fly heads were separated and fixed in 4% Paraformaldehyde (EM grade, Polysciences Inc.) in 1× PBHS for 30 minutes at RT. Then the proboscis was removed and the heads were fixed for an additional 5 minutes at RT. The brain was finally dissected and washed in 1× PBS 3 times for 30 minutes each. The brains were mounted on a coverslip with Vectashield mounting media with DAPI (VectorLab) and observed with an Olympus FV100 confocal microscope. DAPI- stained cell nuclei were visualized at 405 nm, Texas red Dextran at 633 nm.

### Statistical analysis

Statistical analysis was performed by one-way or two-way ANOVA and Bonferroni multiple comparison *post hoc* test. Statistical analyses were performed with Statview (Adept Scientifics, Bethesda, MD) or GraphPad Prism5.
